# Innovative approaches in lung tissue engineering: the role of exosome-loaded bioscaffolds in regenerative medicine

**DOI:** 10.3389/fbioe.2024.1502155

**Published:** 2024-12-20

**Authors:** Mohammad Torkashvand, Leila Rezakhani, Zahra Habibi, Abdolhamid Mikaeili, Shima Rahmati

**Affiliations:** ^1^ College of Engineering, University of Tehran, Tehran, Iran; ^2^ Fertility and Infertility Research Center, Health Technology Institute, Kermanshah University of Medical Sciences, Kermanshah, Iran; ^3^ Department of Tissue Engineering, School of Medicine, Kermanshah University of Medical Sciences, Kermanshah, Iran; ^4^ Clinical Research Development Unit, Hajar Hospital, Shahrekord University of Medical Sciences, Shahrekord, Iran; ^5^ Medical Biology Research Center, Health Technology Institute, University of Medical Sciences, Kermanshah, Iran; ^6^ Cancer Research Center, Shahrekord University of Medical Sciences, Shahrekord, Iran

**Keywords:** lung regeneration, tissue engineering, exosome-loaded scaffolds, organoids, decelullarization

## Abstract

Lung diseases account for over four million premature deaths every year, and experts predict that this number will increase in the future. The top cause of death globally is diseases which include conditions like lung cancer asthma and COPD. Treating severe acute lung injury is a complex task because lungs struggle to heal themselves in the presence of swelling inflammation and scarring caused by damage, to the lung tissues. Though achieving lung regeneration, in controlled environments is still an ambition; ongoing studies are concentrating on notable progress, in the field of lung tissue engineering and methods for repairing lung damage. This review delves into methods, for regenerating lungs with a focus on exosome carry bioscaffolds and mesenchymal stem cells among others. It talks about how these new techniques can help repair lung tissue and improve lung function in cases of damage. Also noted is the significance of *ex vivo* lung perfusion (EVLP), for rejuvenating donor lungs and the healing properties of exosomes in supporting lung regeneration.

## 1 Introduction

Lung diseases account for over four million premature deaths annually, and their prevalence is expected to rise in the coming years. Chronic respiratory diseases, including lung cancer, asthma, and chronic obstructive pulmonary disease (COPD), together represent the third leading cause of death worldwide (Uthman, 2016). Repair of severe acute lung injury remains extremely challenging due to the lung’s limited ability to regenerate and the disordered environment formed by edema, inflammation, and fibrosis following lung disruption (Ma et al., 2018). Steady cell turnover helps the mature mammalian lung maintain homeostasis, and diverse cell populations are necessary for lung remodeling and renewal (Kotton and Morrisey, 2014). But following an injury, a specific subset of facultative lung progenitors is triggered to initiate remodeling, a process that repairs the damaged tissue. When this mechanism is disrupted, healing fibrosis develops, which results in scarring, aberrant lung regeneration, and compromised organ function (Beers and Morrisey, 2011; Wansleeben et al., 2013). For those with end-stage lung failure, lung transplantation (LTX) is still the only available treatment. Due to the limited number of tissue donors and viral contamination, an attempt has been made to construct the tissue using diverse cells and scaffolds, both natural and synthetic. This has resulted in the development of a new concept in science known as tissue engineering. As an academic field, tissue engineering has created a unique opportunity for the invention and refinement of therapeutic procedures for the treatment of congenital and acquired disorders, and its three basic pillars are scaffolds, growth factors, and cells. Scaffolds are a physical support and template for developing cells and tissues (Ashammakhi et al., 2022).

Bioengineered lung tissues are a new field of study that can help with this problem. While creating a fully functional bioengineered lung that might be transplanted is still difficult, new methods are being investigated to create ex vivo-engineered lung tissues that function and have the right gas exchange characteristics (Wagner et al., 2013). Despite advancements in organ preservation methods, particularly *ex vivo* lung perfusion (EVLP), new approaches are desperately needed to close the therapeutic gap in biological (acellular) lung scaffolds and increase the number of transplantable tissues available (Shakir et al., 2022). EVLP is a method that has revolutionized lung transplantation by improving the evaluation and use of donor lungs that might have been considered unfit, for transplant in the past. Before EVLP was introduced into practice numerous donor lungs with potential were rejected due to worries about their state leading to an organ utilization rate of 15–20 percent. Nevertheless, research indicates that approximately 40 percent of these declined lungs could be eligible for transplantation after undergoing further assessment, with EVLP. The importance of EVLP goes beyond evaluating lung viability as it plays a vital part, in revitalizing donor’s lungs that may be, on edge.

Increased Utilization: EVLP has greatly expanded the pool of useable donor lungs by offering a platform for further evaluation, enabling transplant centers to use organs previously thought to be unsuitable.

Decrease in Primary Graft Dysfunction (PGD): While PGD is still a risk, EVLP has been demonstrated to lessen its prevalence by enabling improved donor lung selection based on physiological data collected in real-time rather than only pre-transplant evaluations.

Identifying Biomarkers: EVLP offers the chance to find biomarkers that are indicative of post-transplant results, which will allow donor selection procedures to be further improved.

Therapeutic Interventions: According to recent research, pharmacological medicines or antibacterial therapies that are intended to enhance lung function before transplantation may be given during EVLP.

**FIGURE 1:**
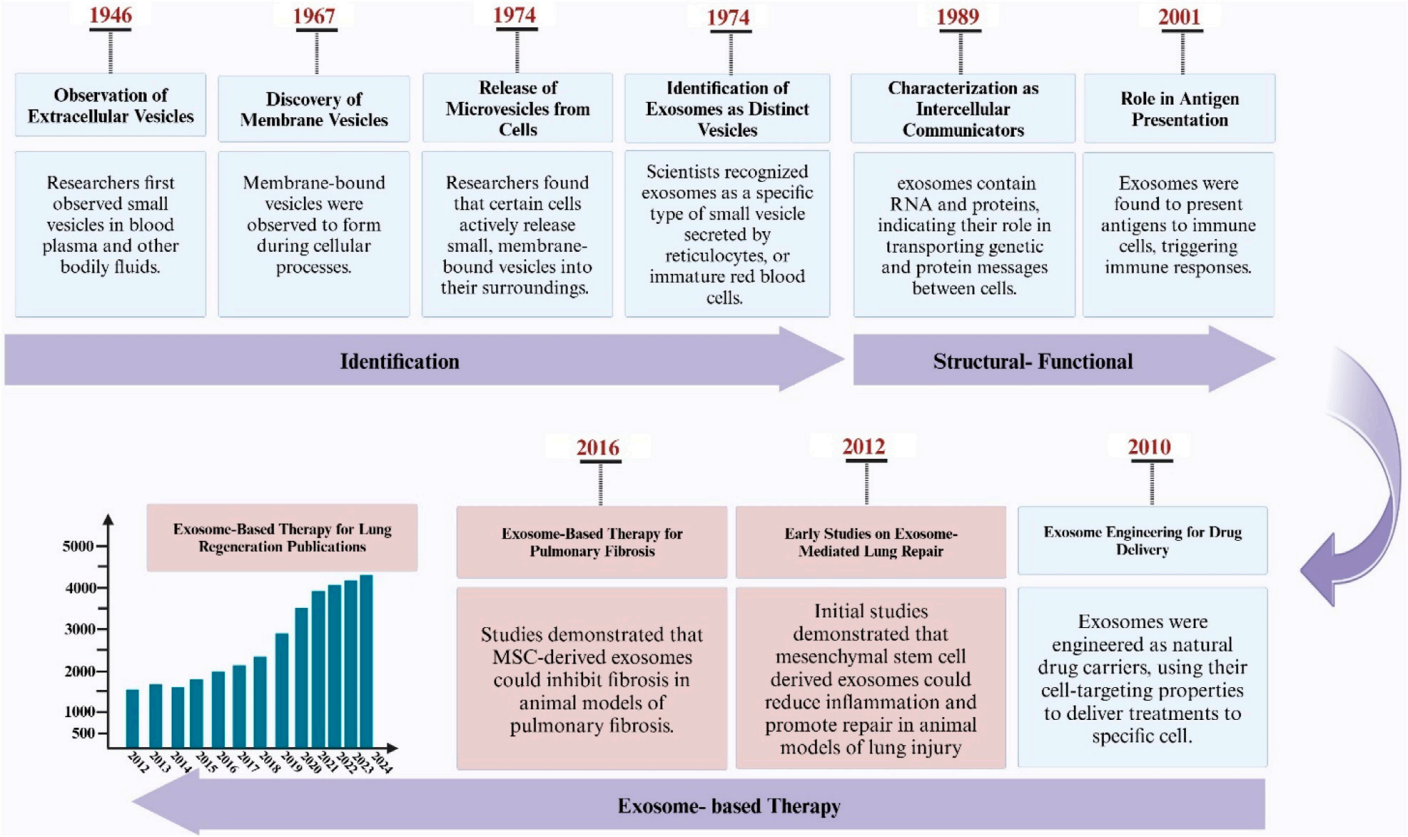
Illustration depicting the discovery timeline of exosomes, their application milestones in pulmonary research, and a PubMed-derived graph showing the increasing trend of exosome-related publications in lung studies.

Exosomes are nanovesicles that carry bioactive molecules and play an important role in cell-to-cell communication (Record et al., 2014). They have important characteristics that make them superior to biological barriers and thus have a direct effect on various physiological and pathological processes (Rajput et al., 2022). Based on research, it has been determined that exosomes can be an effective mechanism in tissue regeneration, especially in lung tissue regeneration (Chen and Cai, 2023; Hong et al., 2019; Sen et al., 2023). Also, in research, exosomes that are derived from certain cells or tissues have been investigated as a cell-free approach for the treatment of lung diseases, which promotes tissue repair and regeneration by promoting interactions between different lung cell lineages and facilitating paracrine-mediated bioburden transport (Liu et al., 2019; Thomas et al., 2022; Zhang et al., 2019). For this reason, investigating the mechanisms of using exosomes as potential therapeutic agents for lung regenerative medicine is particularly important in tissue engineering and regenerative medicine (Hade et al., 2021). In recent years, the use of exosomes in the clinic has increased significantly (Figure 1).

## 2 Bio-scaffolds

### 2.1 Bioactive exosome-loaded scaffolds

Exosomes, also known as extracellular vesicles, play critical roles in a variety of biological processes, including cell proliferation, differentiation, and survival (Mantile et al., 2021). They originate from a variety of sources, including stem cells and immune cells, and are enclosed in a bilayer membrane to safeguard their genetic material and proteins before being delivered to target cells (Hong et al., 2019). Exosome-containing proteins include membrane transport proteins, tetraspanins, biogenesis-related proteins, and heat shock proteins (Vojtech et al., 2019). Scaffolds enriched with exosomes promise to be a potential strategy in regenerative medicine, particularly for lung tissue engineering (Antich-Rosselló et al., 2021). These scaffolds mix exosomes, which are extracellular vesicles necessary for intercellular communication, with biomaterial scaffolds to aid in tissue repair and regeneration (Re et al., 2021; Rahmati et al., 2023). The ability of exosome-loaded scaffolds to overcome the limitations of traditional cell therapies, like immune system rejection and decreased cell survival, is one of its key benefits (Gu et al., 2021).

**FIGURE 2:**
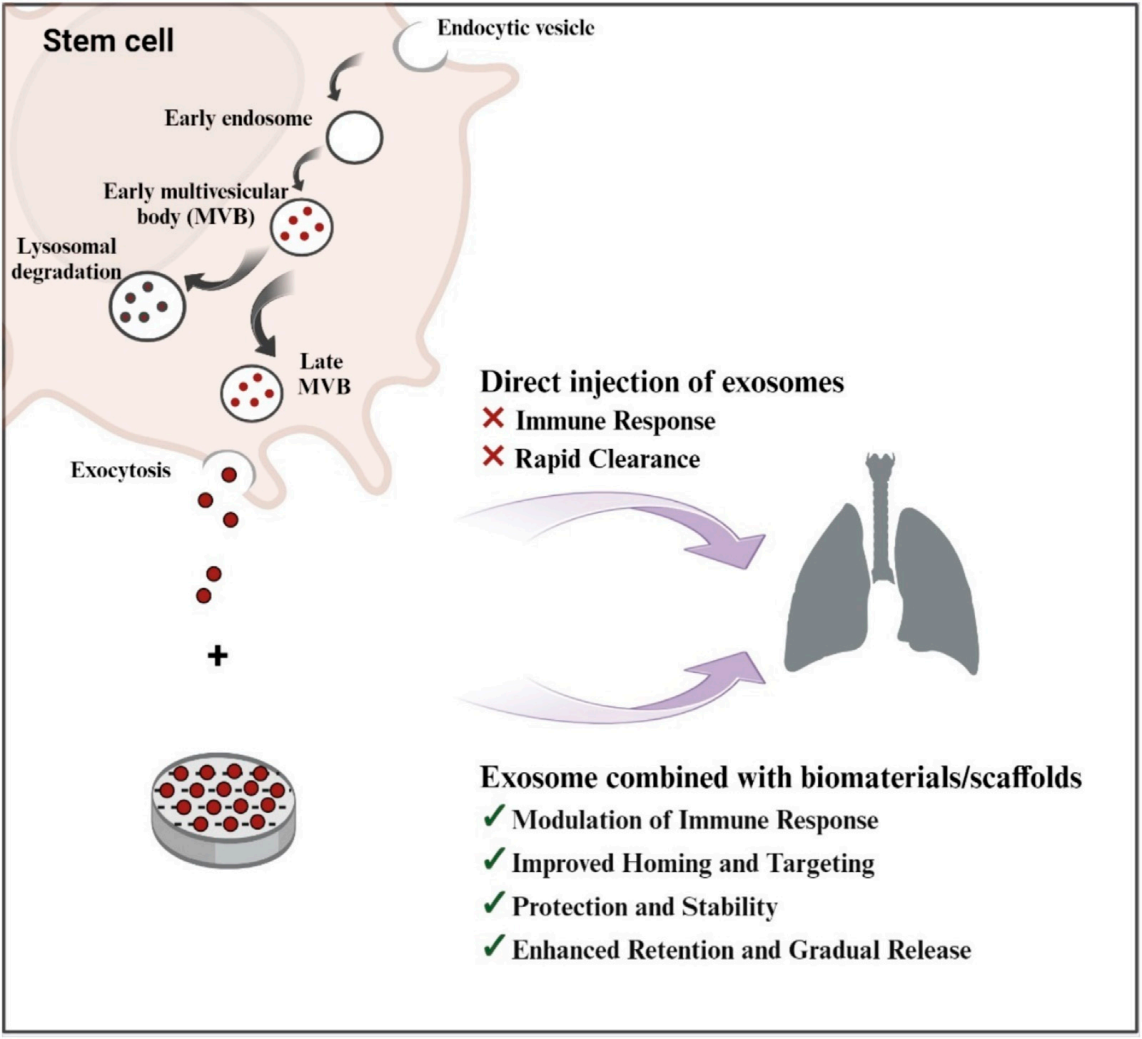
Schematic Illustration of exosome biogenesis and the benefits of exosome-loaded bio-scaffolds for lung regeneration (Created with BioRender.com).

Exosome-loaded bioactive scaffolds, which can be made into three-dimensional structures like bioactive glasses, offer a special cellular environment that can boost exosomes’ therapeutic potential and encourage tissue repair (Poongodi et al., 2021). The use of bioactive scaffolds can improve tissue repair and regeneration by promoting cell adhesion, proliferation, and differentiation (Pacelli et al., 2017) (Figure 2).

To improve proangiogenic activity in bone healing, for instance, exosomes extracted from human bone marrow MSCs activated by dimethyloxaloylglycine were placed onto a porous hydroxyapatite scaffold (Vig and Fernandes, 2022). Similarly, exosomes extracted from human adipose-derived stem cells were combined with poly (lactic-co-glycolic acid) (PLGA)/polydopamine (pDA) structures to create a cell-free bone tissue engineering system (Torrecillas-Baena et al., 2023). Exosomes were released from the scaffold slowly and continuously, which promoted MSC migration and greatly enhanced bone repair (Swanson et al., 2020).

Recent developments have focused on the use of specific biomaterials and scaffolds to stimulate cells to increase exosome production and on the integration of exosomes into 3D scaffolds with different architectures for implantation into damaged tissue (Brennan et al., 2020). Researchers have also developed several methods to sustainably introduce exosomes into the environment after myocardial infarction (Bellin et al., 2019). For example, exosomes produced from cardiomyocyte-derived induced pluripotent stem cells and encapsulated in hydrogel patches were directly delivered into infarcted rats’ hearts (Cheng et al., 2021). Exosome patches increased ejection fraction, avoided cardiomyocytic hypertrophy, decreased ischemic injury, and improved heart repair (Ping et al., 2023). Bioactive scaffolds can enhance tissue repair and regeneration by stimulating cell adhesion, proliferation, and differentiation. Bioactive scaffolds can enhance tissue repair and regeneration by stimulating cell adhesion, proliferation, and differentiation (Stratton et al., 2016). Wang et al. discovered that stem cell transplants with a biomimetic scaffold promote lung recovery. To alter lung tissue, they used a scaffold loaded with exosomes from mesenchymal stem cells (MSC) (Wang et al., 2023). Exosomes were able to recruit and differentiate MSCs, resulting in the production of functional lung tissue (Wang et al., 2023).

Research by Yang and El Haj (2006) also highlighted the importance and value of designing and manufacturing scaffolds for cell and drug transfer, recognizing the need for biocompatible and biodegradable materials (Yang and El Haj, 2006). Huang et al. (2021) also discussed the practical importance of exosome-filled scaffolds in the tissue regeneration and repair process and emphasized the important role of exosomes in cell communication and tissue regeneration (Huang et al., 2021). Additionally, Goldberg investigated most nanostructured materials for biotechnology and drug delivery and suggested using them to create exosome-loaded structures (Goldberg et al., 2007).

#### 2.1.1 Role of exosomes in donor lung regeneration

The therapeutic potential of exosomes in lung regeneration is supported by several studies demonstrating their ability to promote tissue repair, reduce inflammation, and enhance cellular function (Xie and Zeng, 2020). Research have shown that exosomes derived from lung spheroid cells (LSC-Exo) exhibit superior therapeutic benefits compared to those derived from mesenchymal stem cells (MSC-Exo) in models of pulmonary fibrosis (Dinh et al., 2020). Inhalation treatment with LSC-Exo significantly attenuated fibrosis induced by bleomycin and silica, restoring normal alveolar structure and decreasing the buildup of collagen and the growth of myofibroblasts appears to be a result of using exosomes in combating lung fibrosis. Moreover, exosomes have been shown to have inflammatory properties by influencing immune responses positively. In conclusion, Exosomes present an approach, to therapy with prospects, for improving the restoration of donor lungs. Their capability to enhance cell viability diminish inflammation and aid in tissue healing makes them crucial contributors to forthcoming approaches for lung ailments. Further exploration into the mechanisms and uses of exosomes will be crucial for creating treatments targeted at enhancing results for individuals, with impaired lung functionality.

Mesenchymal stem cell-derived exosomes can inhibit lung cancer cell proliferation and epithelial-mesenchymal transition (Wang et al., 2022). They could be achieved by delivering active cargoes such as miRNAs that directly target specific genes involved in tumor progression, such as MIER3 (Xiao-Ni et al., 2021). Exosomes also regulate processes such as cell movement, cell proliferation, cell phenotype, and cell growth (Xu et al., 2018). They have properties such as anti-angiogenic and anti-inflammatory, which significantly affect the tissue regeneration process (Olejarz et al., 2020). In addition, they can transport active miRNA and release anticancer drugs, which inhibit the growth of tumor cells and cause cell death (Fernandes Ribeiro et al., 2013).

One of the challenges associated with exosome-based bioscaffolds for lung tissue regeneration is the need to improve large-scale production of exosomes to overcome limitations including short cycle time and low targeting capacity (Lu et al., 2023). In addition, another important challenge is the stability and storage conditions of exosomes, which should be taken into account for their correct conversion into the clinical setting (Ana et al., 2021). It is important to closely monitor the biodistribution and pharmacokinetics of exosomes due to factors such as their size and diversity (Choi et al., 2021). Finally, engineering alpha-6 integrin-expressing exosomes to target lung epithelial cells the incorporation of tissue-specific components into exosomes can improve their targeting ability and thereby affect transmigration (Fedele et al., 2015).

Exosomes have important clinical applications, including the potential to provide important information in the early detection of lung cancer (Hu et al., 2020). They also have important properties that make them promising therapeutic agents for diseases such as lung cancer because MSCs as well as their exosomes offer advantages for regenerative medicine such as immunosuppressive and non-immunogenic effects (Reclusa et al., 2017). Exosome-loaded scaffolds have also been thoroughly investigated in research for lung tissue regeneration and repair (Su et al., 2021; Wang et al., 2024; Xie et al., 2021; Yan et al., 2020). Exosomes can also regulate the activity and proliferation of osteoclasts and make them suitable for the tissue regeneration process (Qin et al., 2016).

### 2.2 Cell-based scaffolds in lung regeneration

**FIGURE 3:**
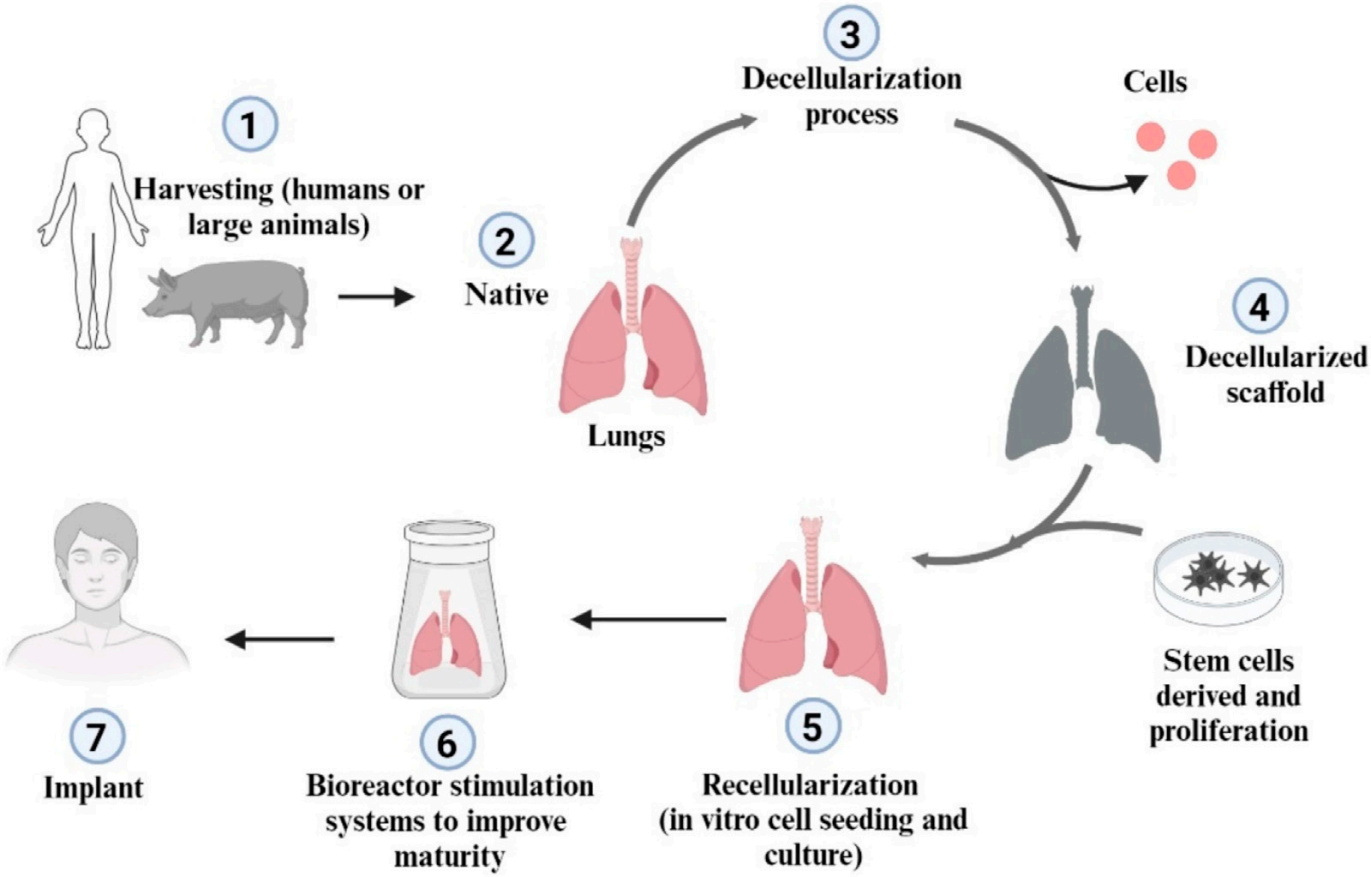
Schematic representation of lung decellularization and recellularization approach (Created with BioRender.com).

Cell-based scaffolds represent a new approach to tissue engineering that integrates cells into biodegradable and biocompatible scaffolds to mimic the extracellular matrix (Edgar et al., 2016). This mimicry provides structural support and biological, chemical, and mechanical signals that influence the development of new tissue (Barnes et al., 2017). Although cell-based scaffolds have shown promise in several applications, including lung regeneration, they also present challenges, such as the need for a reliable source of human-sized scaffolds and a definitive sterilization method (Tapias and Ott, 2014). Figure 3.

Furthermore, stem cell transplantation based on biomimetic scaffolds has been shown to promote lung regeneration (Willie et al., 2010). A three-dimensional artificial MSC implant based on a biomimetic scaffold was constructed to create a favorable regenerative environment for lung tissue (Wang et al., 2023). This approach could help overcome the problems associated with the shortage of organ donors and avoid the need for lung transplantation (Van Raemdonck et al., 2009). However, further research is needed to overcome the challenges associated with cell-based scaffolds, such as the need for a reliable source of human-sized scaffolds and a method for the final sterilization of the scaffolds (Griffin et al., 2018). Advances in artificial intelligence and stem cell transplantation based on biomimetic scaffolds open promising prospects for the future of lung regeneration (Zhang and Wang, 2019).

Recent advances in lung tissue engineering, including the development of smart biomaterials for scaffolds, and the use of on-chip organizational models to mimic the state of lung tissue, represent their important applications (Bennet et al., 2021; Kim et al., 2019). The development of smart biomaterials for scaffolds has also had a significant impact on the field of lung regenerative medicine (Aiman et al., 2022). These biomaterials actually activate certain tissue properties and can respond appropriately to changes in physiological conditions or external stimuli, in the same way that they have been used in various applications, including lung tissue repair and regeneration (Brovold et al., 2018). For example, Balestrini et al. (2015) looked at removing cells from pig pleural membranes to create a tissue engineering alternative to lung tissue repair (Balestrini et al., 2015). In another study by Kim et al. (2019), 3D artificial mesenchymal stem cell transplantation based on a biomimetic scaffold was used to create a positive regenerative niche for *in situ* lung regeneration from endogenous stem cells (Kim et al., 2019).

Lab-on-a-chip models are useful tools for simulating lung tissue conditions, especially in lung reconstructive medicine (Nichols et al., 2014). These models combine microfluidic systems and the cellular microenvironment to create constructs that mimic the structure and function of human organs and tissues while preserving dynamics (Li K. et al., 2019). Lung-on-a-chip models are especially used to summarize lung function at the level of organs in laboratory conditions, where this feature allows lung-on-a-chip models to simulate the special microenvironment of the lungs (Wang et al., 2021). Lung diseases such as cancer, lung infections, asthma, and pulmonary edema can be studied using lung-on-a-chip models (Jadhav et al., 2021). In addition, bionic lungs, which are self-assembled three-dimensional functional units, provide another application for mimicking lung tissue conditions, helping to study diseases and evaluate drug efficacy (Barreiro Carpio et al., 2021). Cell removal techniques were also used to manufacture bioprostheses (Do et al., 2022). The goal of this approach is to create patient-specific lungs for transplantation using cells derived from induced pluripotent stem cells (Wilkinson et al., 2017).

## 3 Hydrogel-based exosome delivery system

ECM of the lungs has been mimicked by artificial polymeric (Doryab et al., 2019). Scaffolds are crucial for tissue engineering (TE) development because they may be customized to imitate cellular microenvironments and provide structural and physicomechanical support, which can be used to moderate cellular physiology (Hussey et al., 2018). Three-dimensional macromolecular polymer networks called hydrogels offer a three-dimensional microenvironmenthat improves exosomes’ therapeutic potential (Frith et al., 2010). They have some viscosity and elasticity and can be created chemically or physically through the use of cross-linking agents (Lim et al., 2020). The use of hydrogels in biomedical applications have attracted a lot of attention because of their structural and mechanical similarities to numerous tissues (Maiz Fernández, 2021). A novel method of tissue engineering, especially for lung tissue regeneration, is represented by hydrogel-based exosome delivery devices (Xie et al., 2022). Exosomes are released by these systems in a regulated and controlled manner to maximize efficacy and reduce side effects. Exosomes are tiny extracellular vesicles that are loaded with certain lipids, proteins, and nucleic acids (Di Bella, 2022). They play a significant function in regulating intercellular communication and are exploited as transport vesicles for therapeutic applications (Cortes-Galvez et al., 2023). However, the stability and destiny of exosomes offer a substantial barrier to therapeutic uses, as they are degraded by the immune system immediately after injection *in vivo* (Ren et al., 2016). Therefore, the delivery method should be optimized to achieve high therapeutic efficacy and specificity, which means delivery of the desired exosomes to the target tissue (He et al., 2022). Biomaterials such as hydrogels allow exosomes to overcome poor tissue retention and provide a platform for controlled release to localize their activity (Pan et al., 2022).

### 3.1 Gelatin scaffold

Gelatin-based scaffolds have shown great promise in lung tissue engineering, as they mimic the natural ECM of lung tissue very well (Doryab and Schmid, 2022). These scaffolds, consisting of a gelatinous matrix, promote the growth and development of lung cells and therefore reflect the cellular microenvironment (Mahfouzi et al., 2021). This property makes gelatin-based scaffolds an ideal platform for research and the potential treatment of lung diseases (Bridge et al., 2018).

One way to improve the therapeutic potential of these scaffolds is to incorporate exosomes into the gelatinous matrix (Kang et al., 2023). Once implanted, these exosomes can be gradually released and provide cells with a continuous supply of nutrients and growth factors (Han et al., 2023). This method is successfully used to regenerate bone tissue, demonstrating the potential utility of gelatin-based scaffolds in tissue engineering beyond lung tissue (Guillén-Carvajal et al., 2023).

**FIGURE 4:**
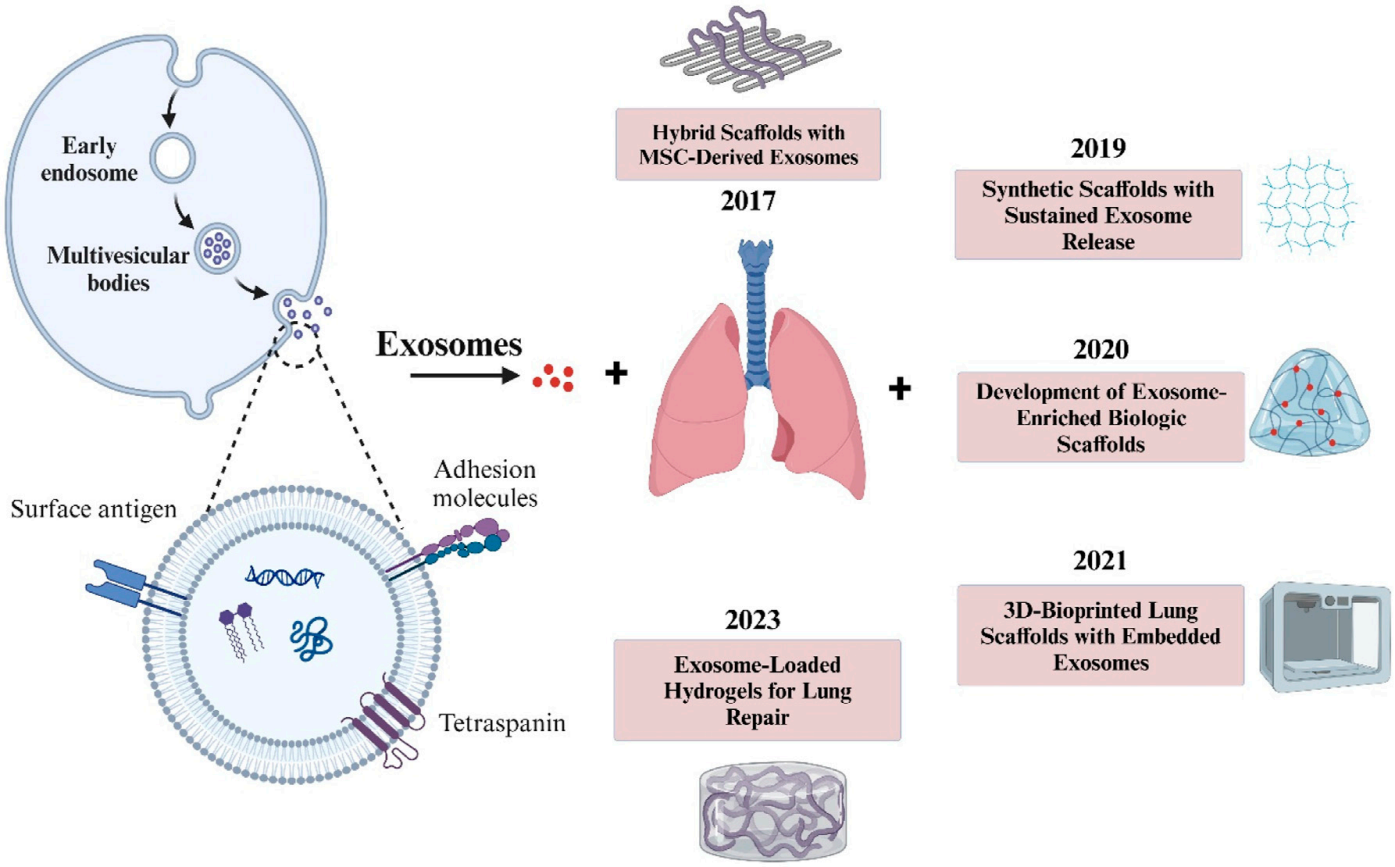
The illustration highlights key stages in exosome formation, from endocytosis to release, alongside notable advancements in scaffold-based lung therapies. This timeline underscores the evolving role of exosomes in tissue engineering for respiratory conditions.

This gelatin scaffold exhibited desirable mechanical and degradation properties, flexibility, and cell adhesion (Stratton et al., 2016). Although gelatin-based scaffolds are very promising, other challenges remain, such as ensuring the long-term stability of the scaffolds, maintaining the integrity of the gelatin matrix, and optimizing the release rate of exosomes (Poongodi et al., 2021). Further research and development are needed to overcome these obstacles and maximize the potential of gelatin-based scaffolds for the treatment of lung diseases. In general, gelatin-based scaffolds, alone or in combination with other materials, are very promising for lung tissue development (Yuan et al., 2022). They have unique properties essential for optimal tissue function and have been shown to promote cell adhesion, proliferation and differentiation (Liu et al., 2022). Current research focuses on refining scaffold design and manufacturing and overcoming the challenges associated with integrating and controlling multiple nozzles in each cell (Giannitelli et al., 2014). Further studies are needed to fully understand the behavior of cells seeded on the scaffold and how they might integrate into the recipient nervous system. More supplementary information is also provided in Table 1 (Figure 4).

**TABLE 1:** Types of bio-scaffolds for regeneration of lung tissue: Materials, fabrication methods, and applications.

Materials in scaffold fabrication	Method of fabrication	Conclusion	Ref.
Decellularized Lung Extracellular Matrix (ECM)	Decellularization using detergents and other reagents	It preserves the complexity of the ECM and offers significant efficiency for lung tissue engineering	Golebiowska et al. (2024)
Synthetic and Natural Polymers	3D bioprinting and organoid techniques	More research is needed on 3D bioprinting, especially to improve epithelialization and vascularization	Yeo et al. (2023)
Acellular Lung Matrices	Recellularization with mesenchymal stem cells	Decellularization of mesenchymal stem cells causes promising lung regeneration *in situ*	Bonvillain et al. (2012)
Lung ECM	Seeded with cells sourced from the transplant recipient	Implantation of recipient cells promotes tissue regeneration and represents a material approach to lung tissue engineering	Ohata and Ott (2020)
Hydrogel Composites	Encapsulation of exosomes in a composite system	Hydrogels act as effective bifunctional carriers in exosomal applications	Xie et al. (2021)
Gelatin	Fabrication of scaffolds using gelatin	Gelatin scaffolds have potential for bone regeneration as a support system and structural material	Echave et al. (2017)
Synthetic Polymers	Electrospinning technique	Electrospun matrices support stem cell differentiation and hold promise for advanced tissue engineering	Flores-Rojas et al. (2023)
Silk Fibroin	Salt-leaching and freeze-drying techniques	Silk fibroin offers remarkable biocompatibility and mechanical strength and is suitable for tissue engineering	Sun et al. (2021)
Chitosan	Fabrication of scaffolds using chitosan	Chitosan scaffolds show suitable biocompatibility and structural properties for tissue engineering	Dong et al. (2020)

## 4 Artificial lung scaffolds

Artificial lung structures are three-dimensional structures designed to mimic the architecture and function of natural lung tissue (Nichols et al., 2011). These structures are extremely important for tissue engineering and lung regeneration (Martins et al., 2007). They can be made from a variety of materials, including synthetic and natural polymers, and are designed to provide structural and physical support while mimicking the cellular microenvironment. One of the most essential aspects of these structures is their ability to renew the lung’s extracellular matrix (ECM) (Jiménez-Gastélum et al., 2019). The ECM is a complex network of proteins, lipids, and carbohydrates that provides structural support, controls cell development and differentiation, and facilitates cell-ECM interactions (Springer, 2019). Artificial lung scaffolds can create an appropriate environment for the growth and development of lung cells by utilizing the ECM function (Romero-López et al., 2017).

**FIGURE 5:**
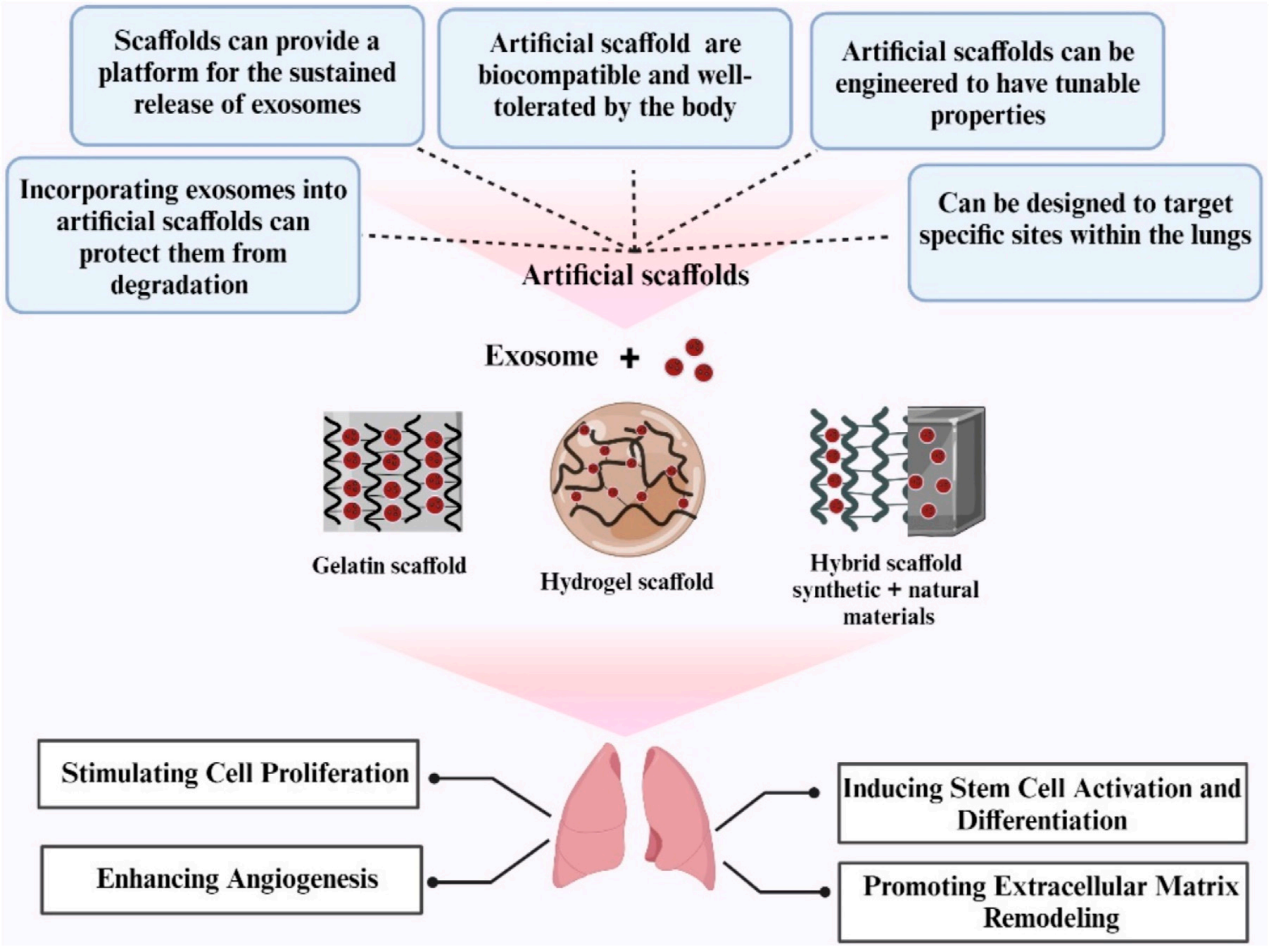
An overview of artificial scaffolds utilized in lung regeneration (Created with BioRender.com).

Despite the potential benefits of artificial lung structures, their development also poses challenges (Nichols et al., 2009). These include the identification of materials that mimic the ECM, provide physical support, and are biocompatible with the body (Qian et al., 2021). Other challenges include integrating cells into structures, maintaining the structural integrity of the structures, and preventing collapse (Turunen et al., 2018). The development of artificial lung scaffolds is hampered by their heterogeneity and potential xenogeneic problems, making it difficult to scale up this technique in a reproducible and regulated manner (Petrella and Spaggiari, 2018). However, artificial or synthetic lung scaffolds, which typically use synthetic and natural polymers, offer an alternative (Okamoto and John, 2013). One of the advantages of using synthetic materials for scaffold fabrication is the ability to tailor their biological and physical properties to achieve a desired scaffold (Turnbull et al., 2018). Despite advances in lung tissue engineering, limitations remain (Kim et al., 2015). These include the complex branched structure of the pulmonary airways and the challenge of poor biocompatibility and haemocompatibility, i.e., the ability to be compatible with living tissue and blood respectively (Bellet et al., 2021). Promising prototypes show that epithelialization and vascularization of grafts can be achieved using different methods (Singh et al., 2016). However, insufficient research has been conducted on 3D bioprinting and organoid techniques for parenchymal lung tissue (Louit et al., 2023). Figure 5.

### 4.1 Hybrid scaffolds

In lung tissue engineering, significant progress has been made in scaffold manufacturing, with a focus on hybrid bioactive scaffolds (Edgar et al., 2016). These new scaffolds use a synthetic beta copolymer membrane with the ECM, creating a unique cellular microenvironment that supports tissue engineering in the lung (De Santis et al., 2018). Hybrid scaffolds are critical for tissue engineering as they provide essential properties such as strength, elasticity, nutrient transfer, and cellular remodeling, all of which are vital for optimal tissue functionality (Wasyłeczko et al., 2020). The use of hybrid materials in tissue engineering scaffolds in the lung is a growing area of research aimed at creating an optimal environment for tissue regeneration (Silva et al., 2010). The challenges and requirements for constructing such scaffolds are being resolved through interdisciplinary collaboration, and ongoing research focuses on perfecting scaffold design and manufacturing (Hollister, 2009). An important innovation in this area is the use of hybrid materials to build an optimized lung structure (Vallet-Regí et al., 2011). In this strategy, the favorable properties of two or more materials are combined to create a final scaffold that overcomes the limitations of each component (Pina et al., 2019). For example, the favorable properties of Acellular scaffolds for cell adhesion sites, organization, and differentiation signals combined with synthetic materials and advanced manufacturing techniques can lead to a desirable lung scaffold (Ratheesh et al., 2017).

Despite the promising potential of these hybrid scaffolds, it must be recognized that they represent an ambitious goal that requires further scientific research and development (Roseti et al., 2017). Ultimately, the goal is to create functional and biocompatible constructs for tissue regeneration, a challenge that must be addressed through in-depth research and experimentation (Leikeim, 2022). Furthermore, the integration of synthetic and natural materials into scaffolds not only promises to revolutionize the field of tissue engineering but also opens new avenues for the treatment of various lung diseases (Kim et al., 2015).

Lee et al. (2011) discovered that hybrid scaffolds that combine the mechanical strength of synthetic materials with the biological activity of natural materials have the potential to create an environment conducive to lung tissue growth and regeneration (Lee et al., 2011). Biodegradable polymers including poly (ε-caprolactone), polyethylene glycol, and poly (lactic-co-glycolic acid) are commonly used in hybrid scaffolds due to their mechanical stability and rapid decomposition. offers control (Lebourg et al., 2008). However, their natural fraction is frequently formed from extracellular matrix proteins and polysaccharides such as collagen, hyaluronic acid, and fibrin, all of which play critical roles in controlling cell activity and tissue integrity (Bindi et al., 2023). Electrospinning and 3D printing were among the advanced fabrication techniques used to fabricate these hybrid scaffolds with a specific microarchitecture and precise porosity (Abel et al., 2020). Although progress is being made in this area, more research is needed to address challenges such as long-term biocompatibility.

In general, over the past 2 decades, regenerative medicine has experienced significant technological advancements. Each milestone has introduced new methods that enhance tissue regeneration, scaffold design, and treatment efficiency. Table 2 outlines the chronological development of key technologies in this field, demonstrating how innovations have evolved to meet the growing demand for more effective and personalized therapeutic options.

**TABLE 2:** Different strategies for exosome application in lung regeneration, and how methods have progressed and diversified.

Year	Technology	Description	Application	Key advantage	Limitations	Ref.
2005–2010	Decellularization	Technique to create cell-free scaffolds from natural tissues	Organ and tissue regeneration	Maintains extracellular matrix (ECM) structure, promoting natural cell growth	Potential immune response, time-intensive process	Ott et al. (2008)
2010–2015	Biological Scaffolds	Development of scaffolds to support cell survival and functionality in tissue engineering	Various tissue repair (e.g., cartilage, skin)	Enhances cellular attachment and growth	Limited control over release of growth factors	Badylak et al. (2011), Loh and Choong (2013)
2015–2020	Exosome Application	Exploration of exosomes as carriers for growth factors and proteins in regenerative applications	Tissue regeneration, especially in skin and neural tissues	Promotes cellular communication, reduces immune response	Limited methods for isolation and mass production	Batrakova and Kim (2015), Bjørge et al. (2018)
2020–2022	Exosome-Loaded Scaffolds	Integration of exosomes within scaffolds to improve tissue repair and functionality	Lung, liver, and cardiac tissue repair	Combines benefits of scaffolds and exosomes, increases therapeutic effectiveness	Complexity in fabrication, higher cost	Gu et al. (2021)
2022–2024	3D Bioprinting	Use of 3D printing to create complex, customized scaffolds with integrated biological agents	Personalized medicine, organ/tissue reconstruction	Enables precision, customization, and scalability	Requires advanced equipment, regulatory challenges	Joshi et al. (2022), Bogala (2022)
2024	AI-Assisted Scaffold Design	Use of AI for designing optimized scaffold structures with personalized properties	Regenerative medicine, organ engineering	Precision, efficiency in design, potential for customization	Data dependency, requires advanced computational resources	Nosrati and Nosrati (2023)

## 5 Development of *ex vivo* transplantation

Transplantation of organs or tissues, outside the body, from a donor organism for evaluation and treatment before being transplanted into a recipient, is known as *ex vivo* transplantation. The process enables the assessment and improvement of organ viability and function before the actual transplant takes place to increase the likelihood of results.

In the field of lung regeneration, procedures like *ex vivo* transplantation are commonly used to evaluate donor lungs that may not meet transplant requirements by using methods such as EVLP (*Ex Vivo* Lung Perfusion). This approach enables professionals to assess the viability and function of these compromised lungs by perfusing them with a solution under controlled conditions outside the body before transplantation is considered further. During EVLP procedures donor lungs are maintained at a temperature. Perfused with a nutrient-rich solution, like the Steen solution, to simulate blood flow. This method aids in improving lung function and evaluating factors, like oxygen levels and flexibility along with the feasibility (Huang et al., 2023). *Ex vivo* transplant methods notably expand the supply of donor lungs by reconsidering organs that were previously rejected for transplantation suitability purposes. Moreover, they allow for interventions during the perfusion stage, like medication administration or tissue repairs which enhance the graft quality before it is implanted into a recipient. The use of *ex vivo* transplant techniques has demonstrated outcomes in enhancing transplant success rates and lowering issues linked to graft dysfunction (PGG). As studies advance in this area, *Ex vivo* methods might develop more by integrating cutting-edge treatments to improve organ retrieval and enhance patient outcomes post-transplantation.

A basic problem confronting the area of tissue engineering is the inability to create perfusable microvasculature networks capable of supporting tissue viability and withstanding physiological forces without leakage (Nichols et al., 2018). In small animal models, whole bioengineered lungs produced on acellular lung scaffolds were transplanted, but the lungs failed due to intravascular coagulation and endothelial barrier dysfunction, resulting in pulmonary edema (Song et al., 2011). There has been no strategy that has allowed for the long-term survival of bioengineered lungs following transplantation (Wang et al., 2023).

*In vivo* gas exchange is the main outcome of lung bioengineering research. Bioartificial lungs should be transplantable like cadaveric donor lungs, as the lung scaffolds maintain the vascular and airway architecture from the alveoli to the hilum. The viability of recellularized bioartificial lungs was first shown in a rat transplantation model (Ott et al., 2010; Petersen et al., 2010).

To prepare for human clinical trials, it is necessary to scale up from small animals to clinically relevant large animals. To achieve this goal, Zhou H and colleagues repopulated pig lung scaffolds with human basal airway stem cells and umbilical vascular endothelial cells, resulting in human-sized bioartificial lung grafts. Using the pulmonary trunk and left atrial appendage as anastomoses, respectively, the pulmonary artery and vein were heterotopically transplanted into these grafts. One hour after surgical implantation, the graft continued to function and tolerate normal pulmonary blood flow (Zhou et al., 2018).

### 5.1 Organoids in lung regeneration

The term “organoid,” meaning “resembling an organ,” was first used by Smith et al., in 1946 to describe an instance of cystic teratoma (Dutta et al., 2017). Organoids are three-dimensional, self-organizing are created by pluripotent or adult tissue stem cells. They provide an *in vitro* model for drug screening and replicate interactions between cells and niches in tissue formation, homeostasis, regeneration, and disease (Lu et al., 2021a). A unique organoid culture system has been developed due to the technological advancements in in vitro culture technology over the past decade. Organoid cultures have now been adapted to cells of many different epithelial lineages (Rosenbluth et al., 2020).

Lung organoids have been successfully generated using adult stem cells, human pluripotent stem cells (hPSCs), embryonic stem cells (ESCs), and induced pluripotent stem cells (iPSCs). However, recent improvements in lung organoid systems have focused on organoid findings, particularly those from distal airway stem/progenitor cells (Lu et al., 2021a).

Many human lung airways are lined with pseudostratified epithelium, which is composed of various cell types including airway basal cells, secretory cells, ciliated cells, tuft cells, and neuroendocrine cells. Basal cells are present throughout the airways of the human lungs, including the small bronchioles with a diameter of 1 mm. These basal cells constitute approximately 30% of the pseudostratified lung epithelium and are firmly attached to the basal lamina. During homeostasis and repair, they can self-renew and produce secretory and ciliated luminal cells (Lu et al., 2021b).

**FIGURE 6:**
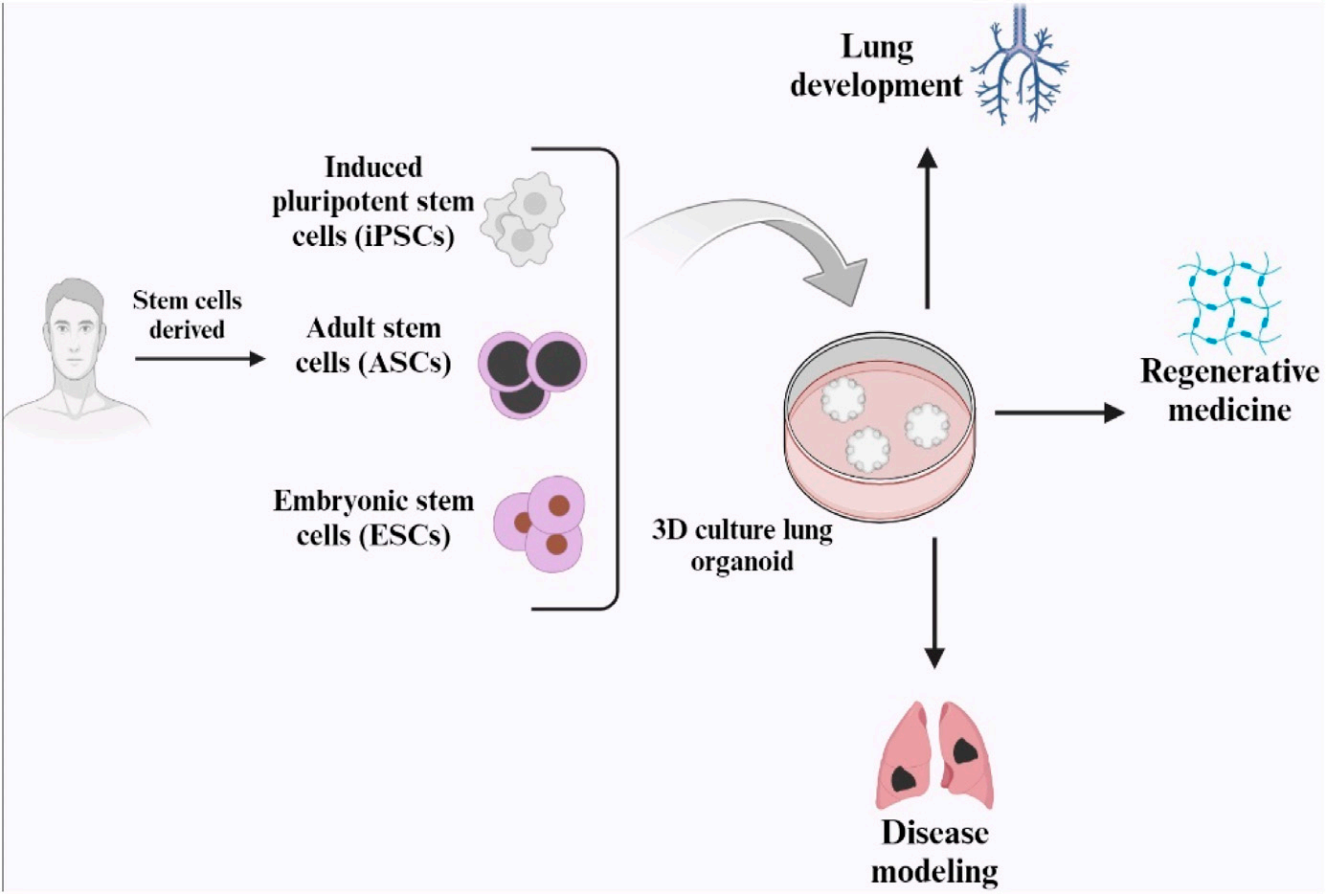
Schematic representation of human lung organoid and its applications (Created with BioRender.com).

Lung organoids can originate from several types of cells: basal cells, alveolar type II cells, and airway secretory cells. The genes that are expressed characteristically in basal cells are Integrin α6, p63, cytokeratin 5 (Krt5), and nerve growth factor receptor (NGFR) (Lu et al., 2021b). The term “tracheosphere” refers to 3D organoids formed from trachea basal cells, and “bronchosphere” refers to organoids derived from bronchi or large airways (Barkauskas et al., 2017) According to Rock et al. (2009a) the first tracheosphere was formed in a week in Matrigel using Krt5-GFPpos murine tracheal basal cells, and it had a visible lumen. After 14 days, p63pos Krt5/Krt14pos basal cells formed the outer layer of a pseudostratified epithelium, whereas Krt8pos ductal secretory cells and ciliated cells formed the inner layer (Rock et al., 2009b) (Figure 6).

## 6 Conclusions and future perspectives

The extracellular matrix of the lungs has various specialized cell types with a distinct architecture that must maintain compliance during respiration, making them difficult organs to engineer. On the other hand, limited production and isolation of exosomes remains a major problem that must be addressed to fully realize the potential of these exosome scaffolds for regenerative medicine (Hade et al., 2021). Furthermore, the short lifetime of exosomes in tissue after *in vivo* implantation remains a major challenge for clinical applications (Mastrolia et al., 2019). One approach to solving this issue is encapsulating exosomes in hydrogels which facilitate their ongoing diffusion into the damaged environment and thus improve their therapeutic effect (Chabria et al., 2021; Tracy et al., 2022). In conclusion, artificial lung constructs and hydrogel-based exosome delivery systems show promise for lung tissue engineering and regeneration (Deng et al., 2023). Types of bio-scaffolds for lung tissue regeneration are summarized in Table 1. The above-mentioned scaffold in this review has numerous applications in lung bioengineering and will lead to advancements in future transplants. However, further research and development are needed to overcome the challenges associated with their use, particularly in terms of material selection and also exosome stability and release (Li X. et al., 2019).

Development of a suitable framework sufficient to fully clarify the physiological and biochemical interactions is necessary before proceeding with the creation of transplantable tissues. Procedures for decellularization and recellularization must also be devised. It is also necessary to study the short- and long-term effects of these transplantable tissues in animal models prior to clinical trials (Taylor et al., 2014). Organoids represent a window of hope for lung regeneration. Lung organoids have proven to be an adaptable and useful tool for studying development, regeneration, homeostasis, and disease. Organoids are created by culturing different cells to represent chronic lung illnesses such as fibrosis and COPD. Future studies using newly separated cells from end-stage sick lungs will considerably increase our understanding of human lung illnesses, allowing for drug development and patient-specific treatment response monitoring.
